# Supplementary figures and images for: Species-Scale Genomic Analysis of Staphylococcus aureus Genes Influencing Phage Host Range and Their Relationships to Virulence and Antibiotic Resistance Genes
Source: mSystems. 2022 Jan 18;7(1):e01083-21. doi: 10.1128/msystems.01083-21 (PMC8765062; doi:10.1128/msystems.01083-21)

A

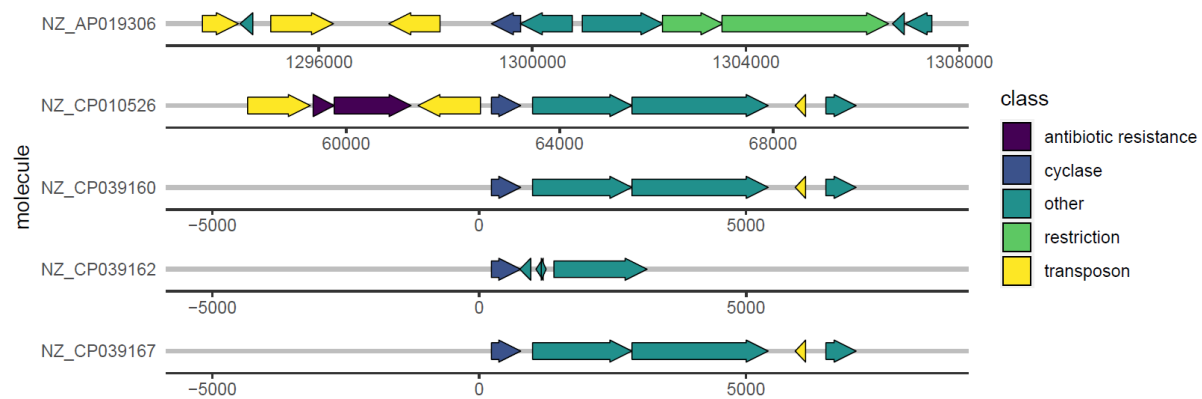

B

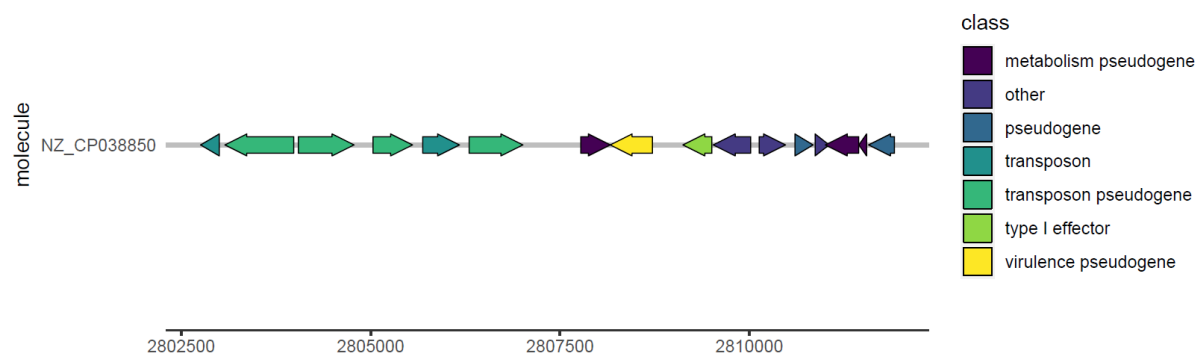

C

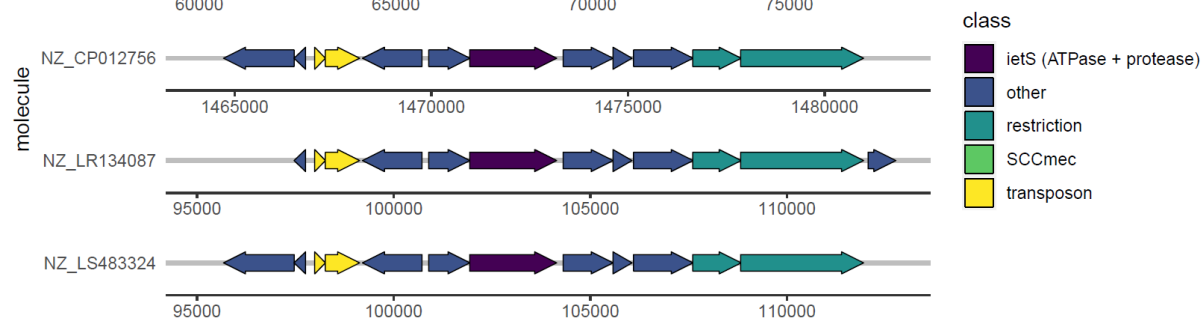

D

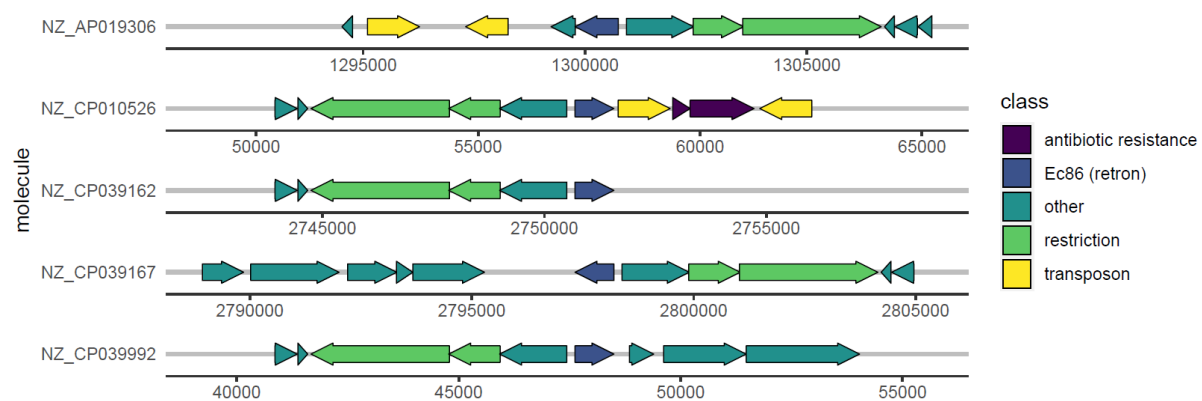

E

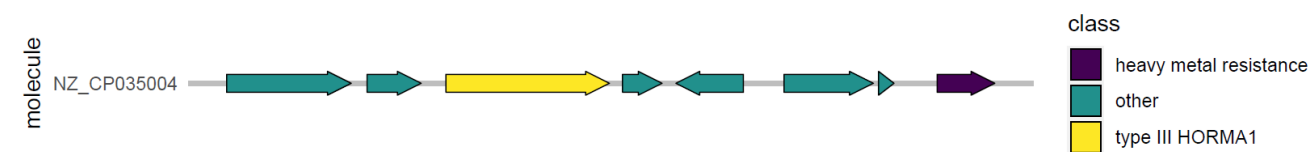

F

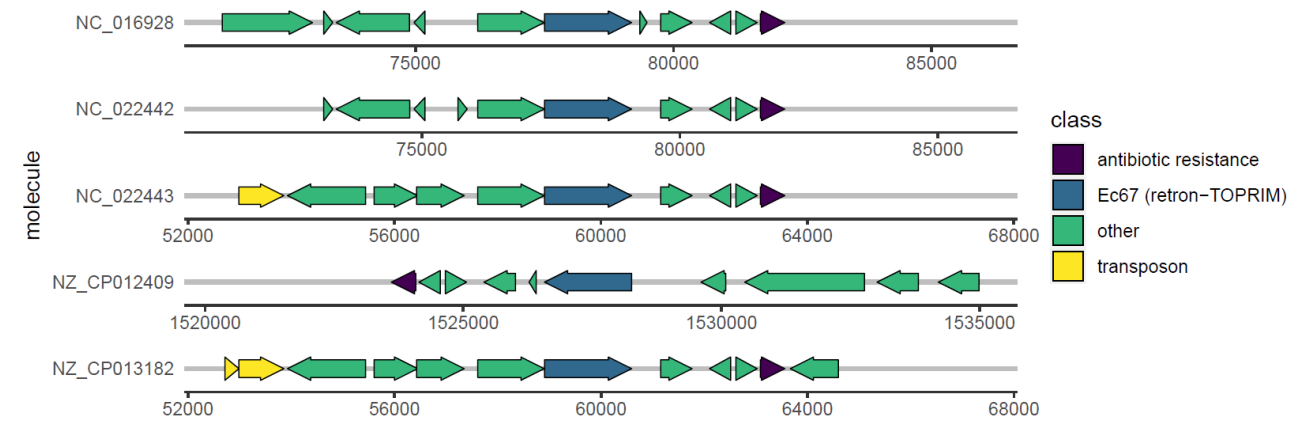

G

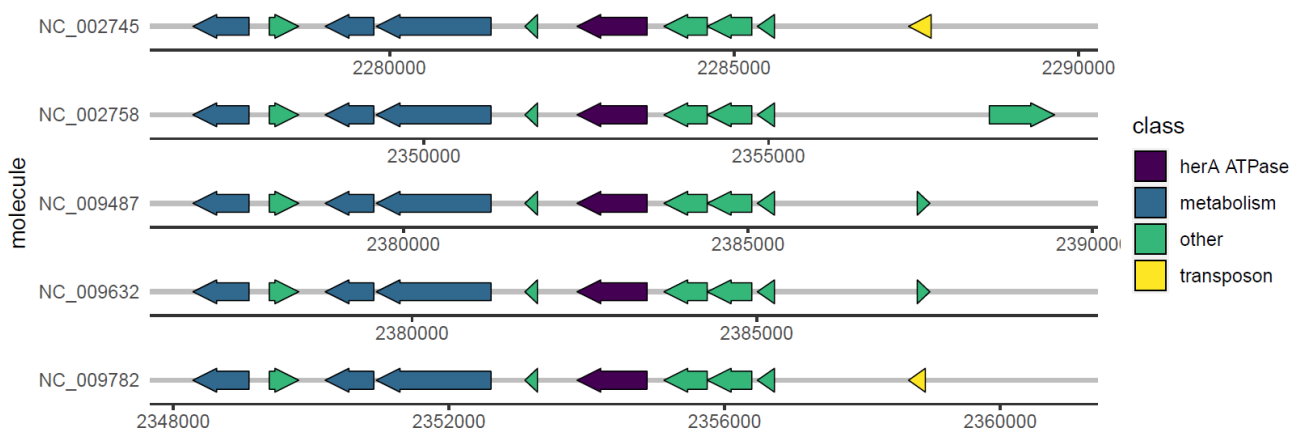

Supplement: FIG S1 [file msystems.01083-21-sf001.pdf]

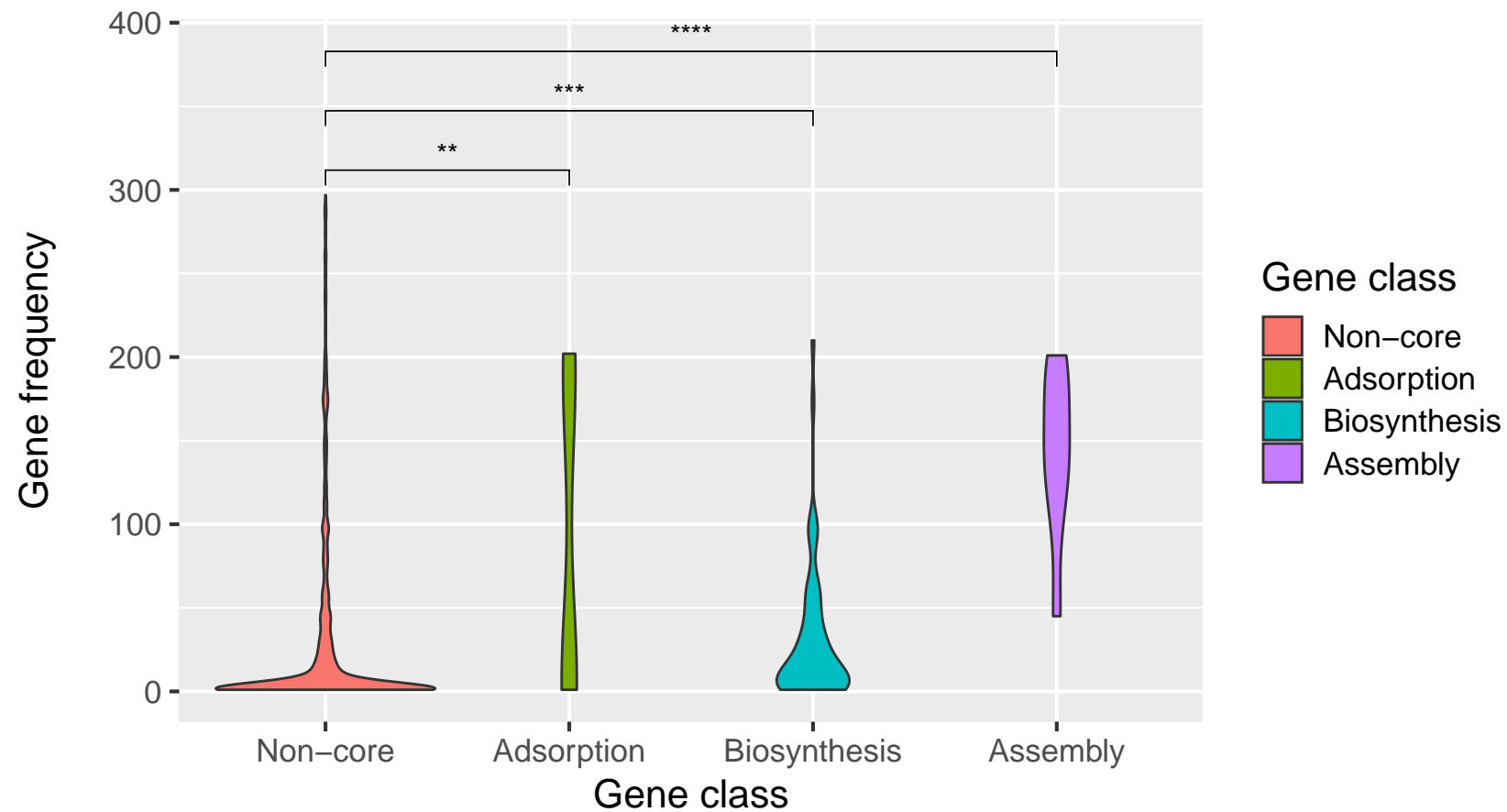

Supplement: FIG S2 [file msystems.01083-21-sf002.pdf]

log(Average number of genes co-encoded)

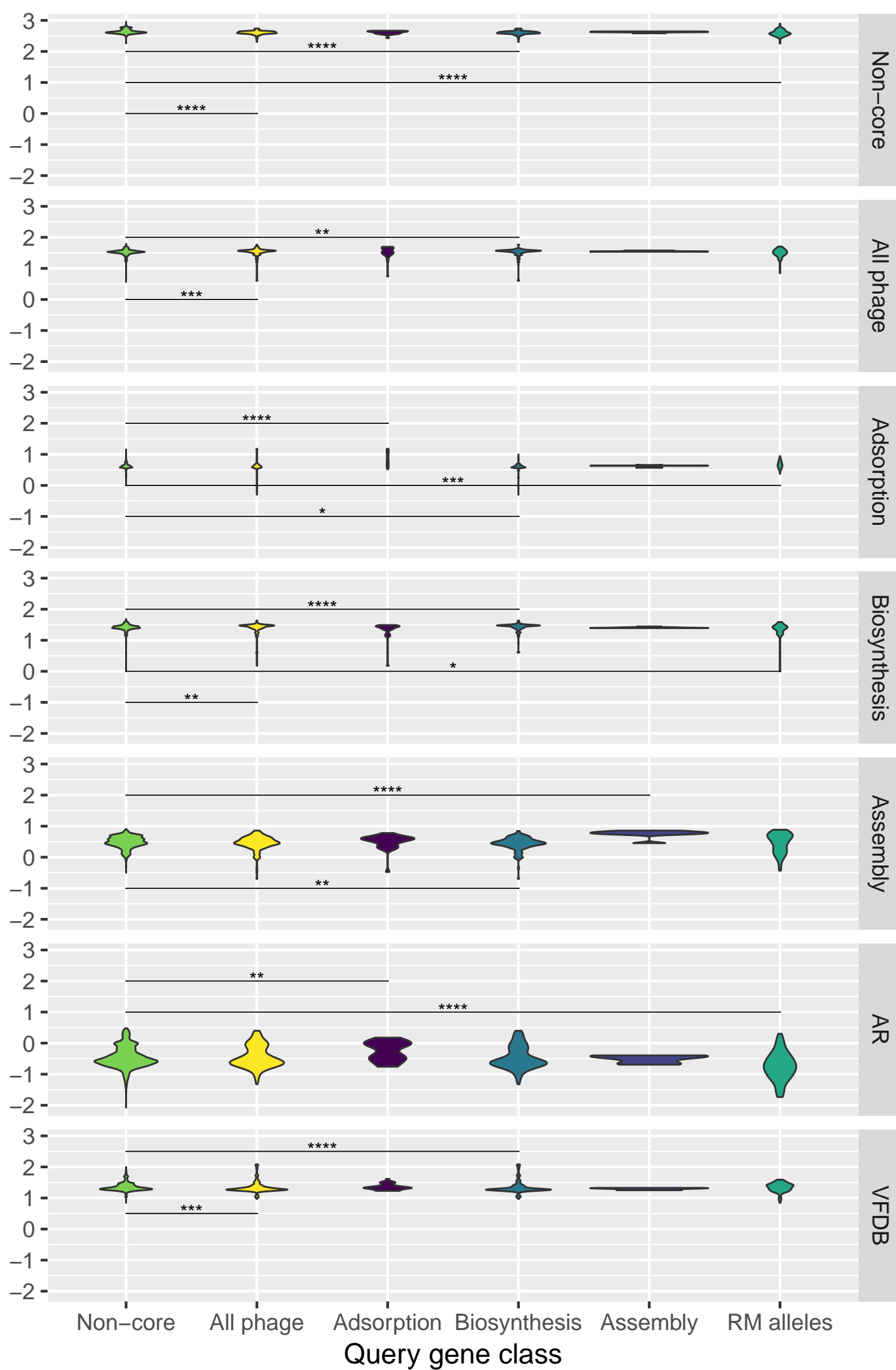

Supplement: FIG S3 [file msystems.01083-21-sf003.pdf]

A

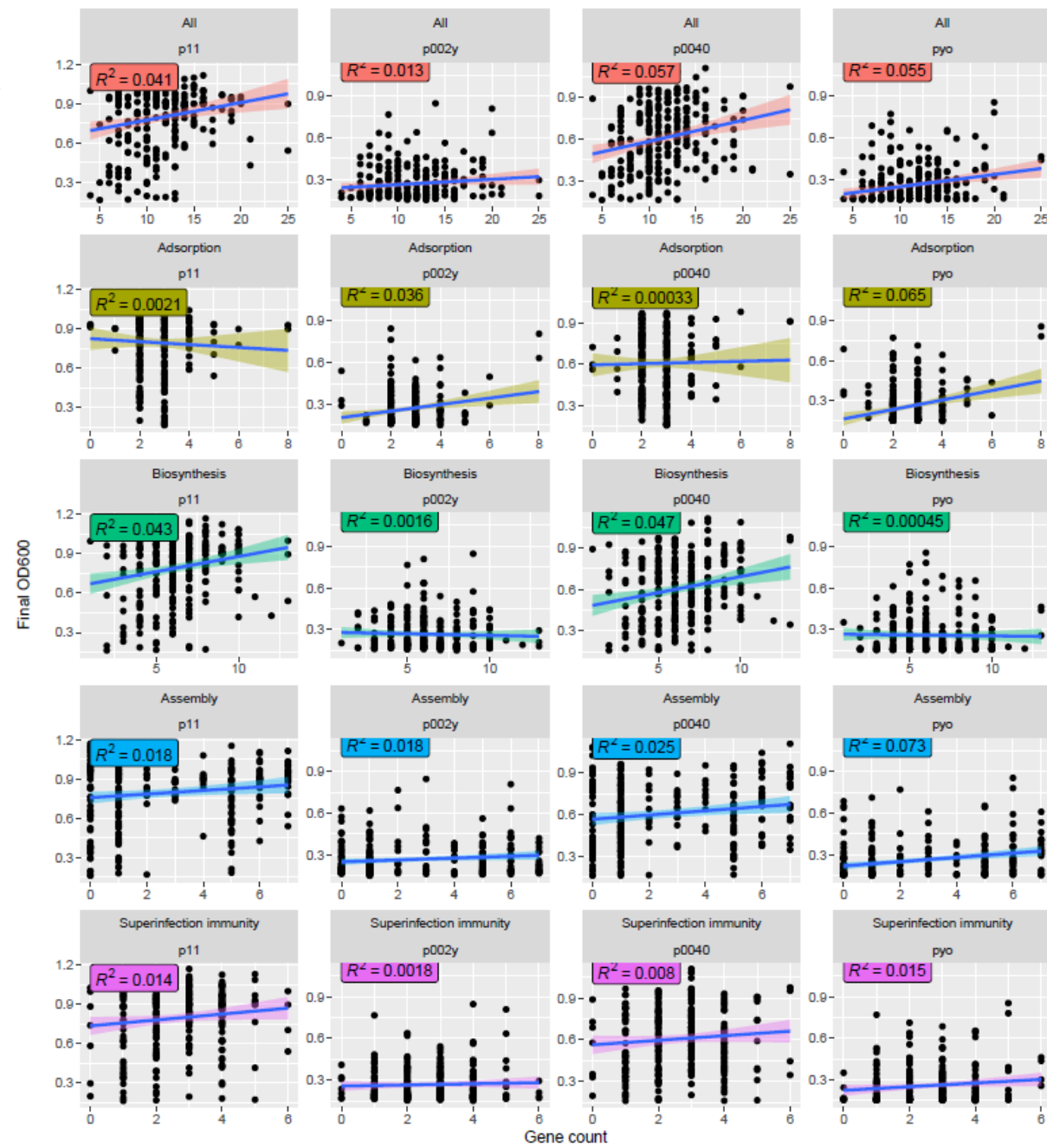

B

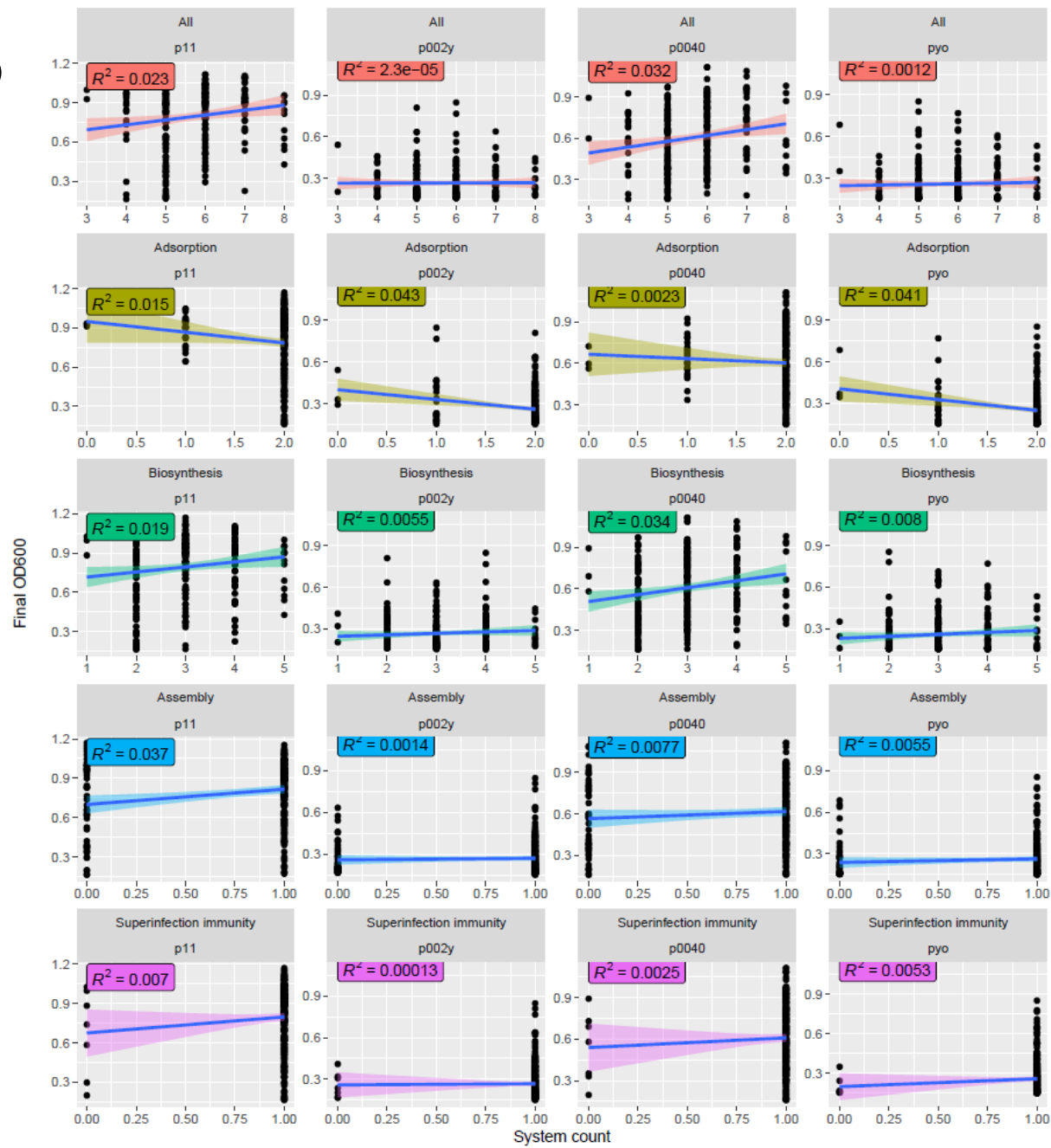

C

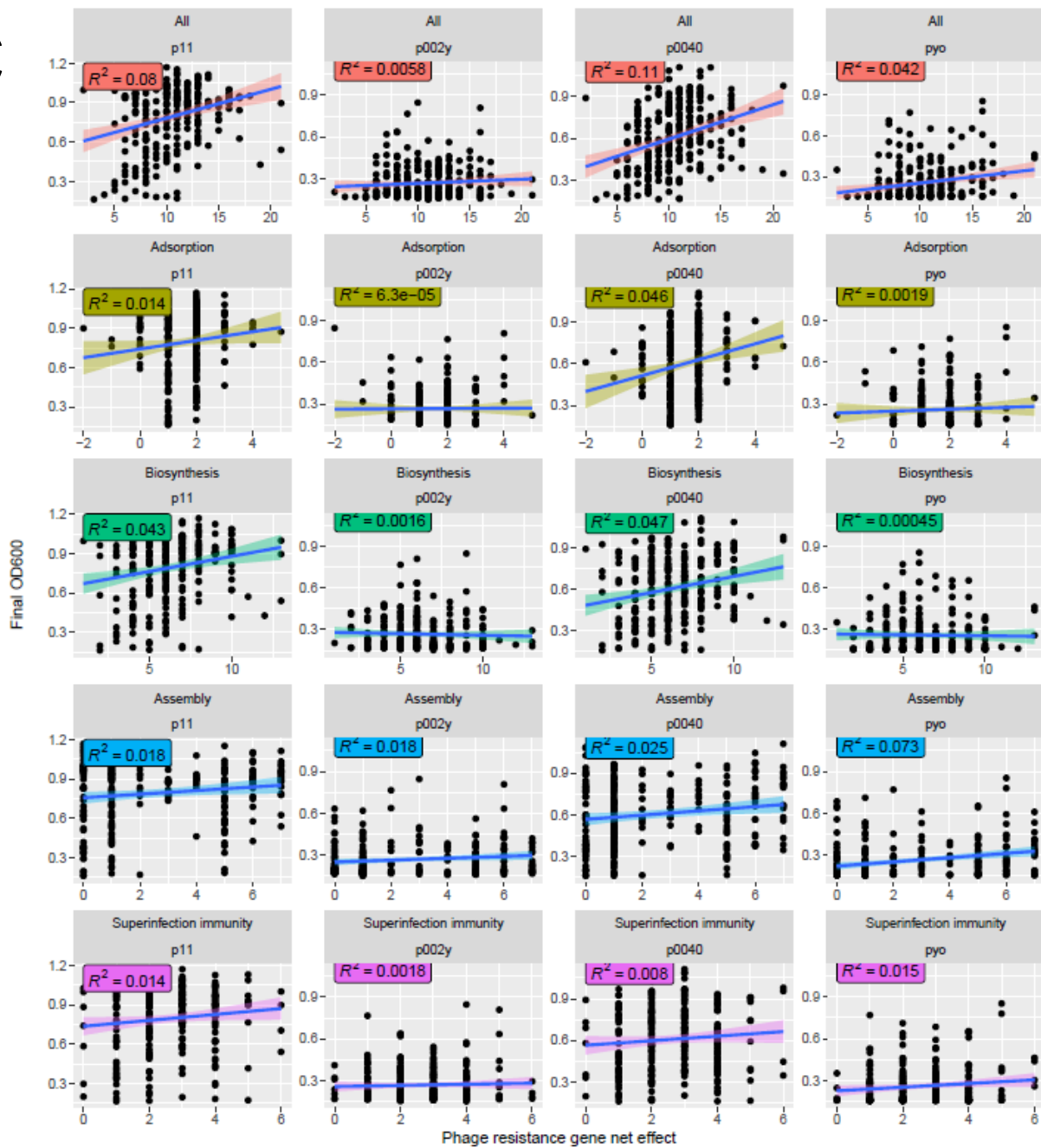

Supplement: FIG S5 [file msystems.01083-21-sf005.pdf]

# A

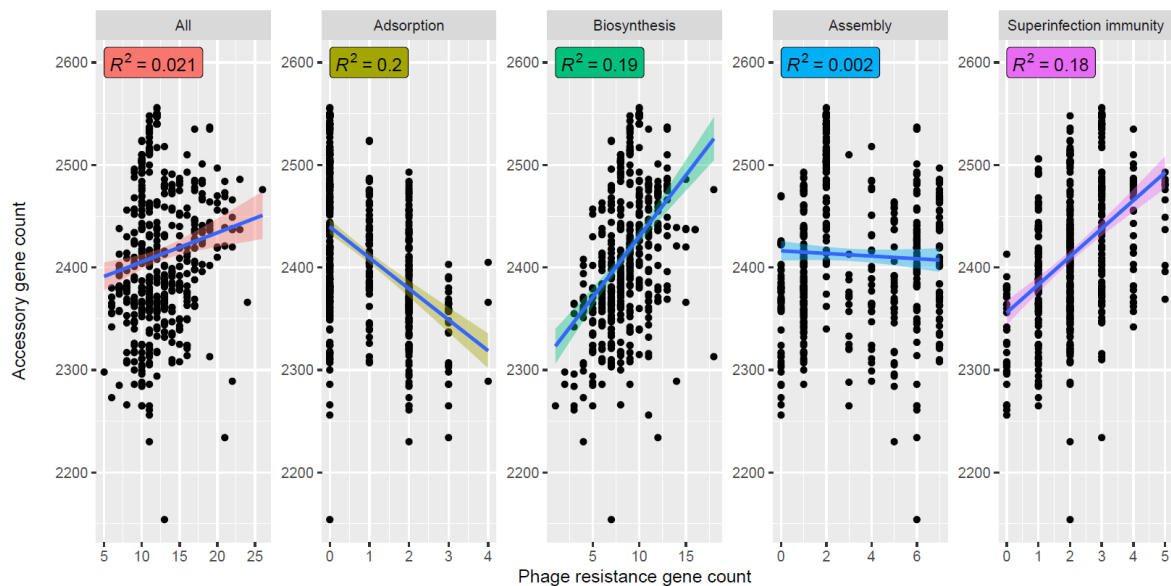

# B

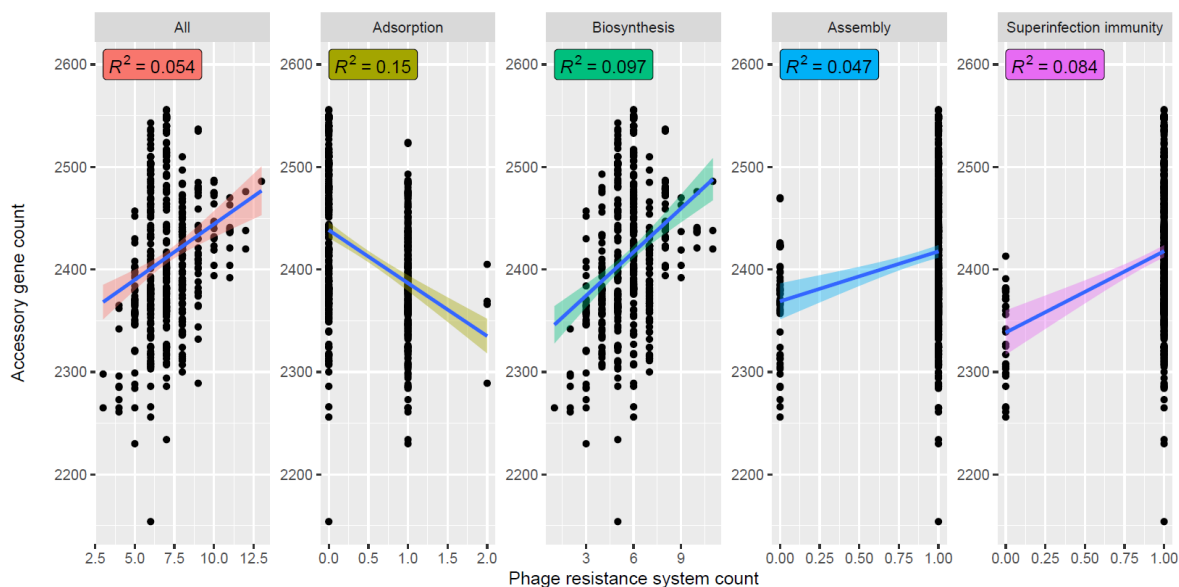

# C

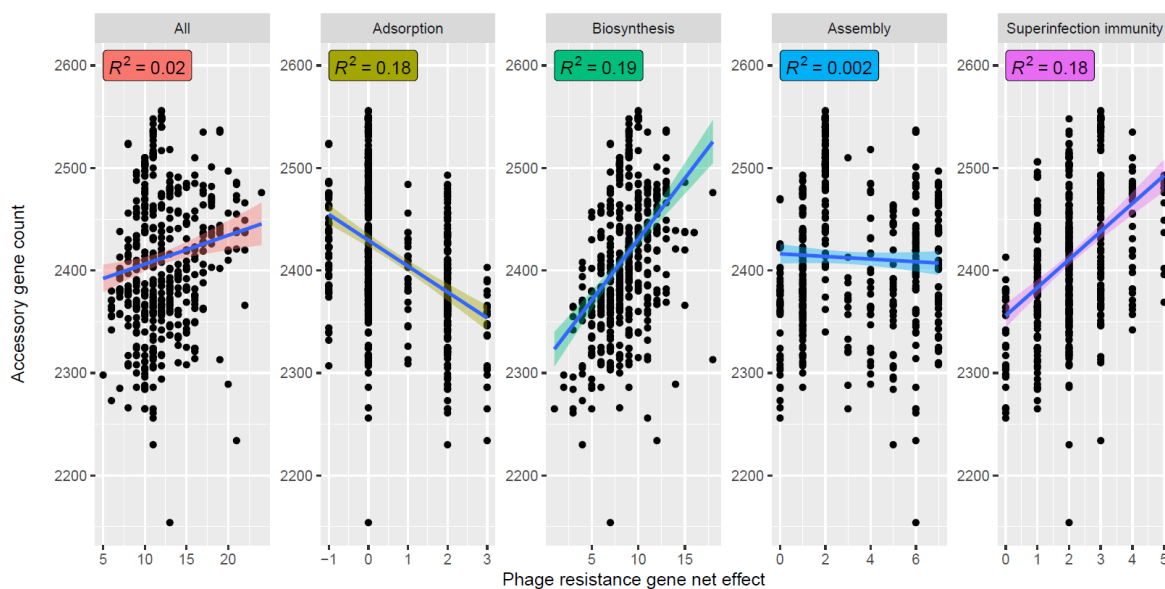

Supplement: FIG S6 [file msystems.01083-21-sf006.pdf]

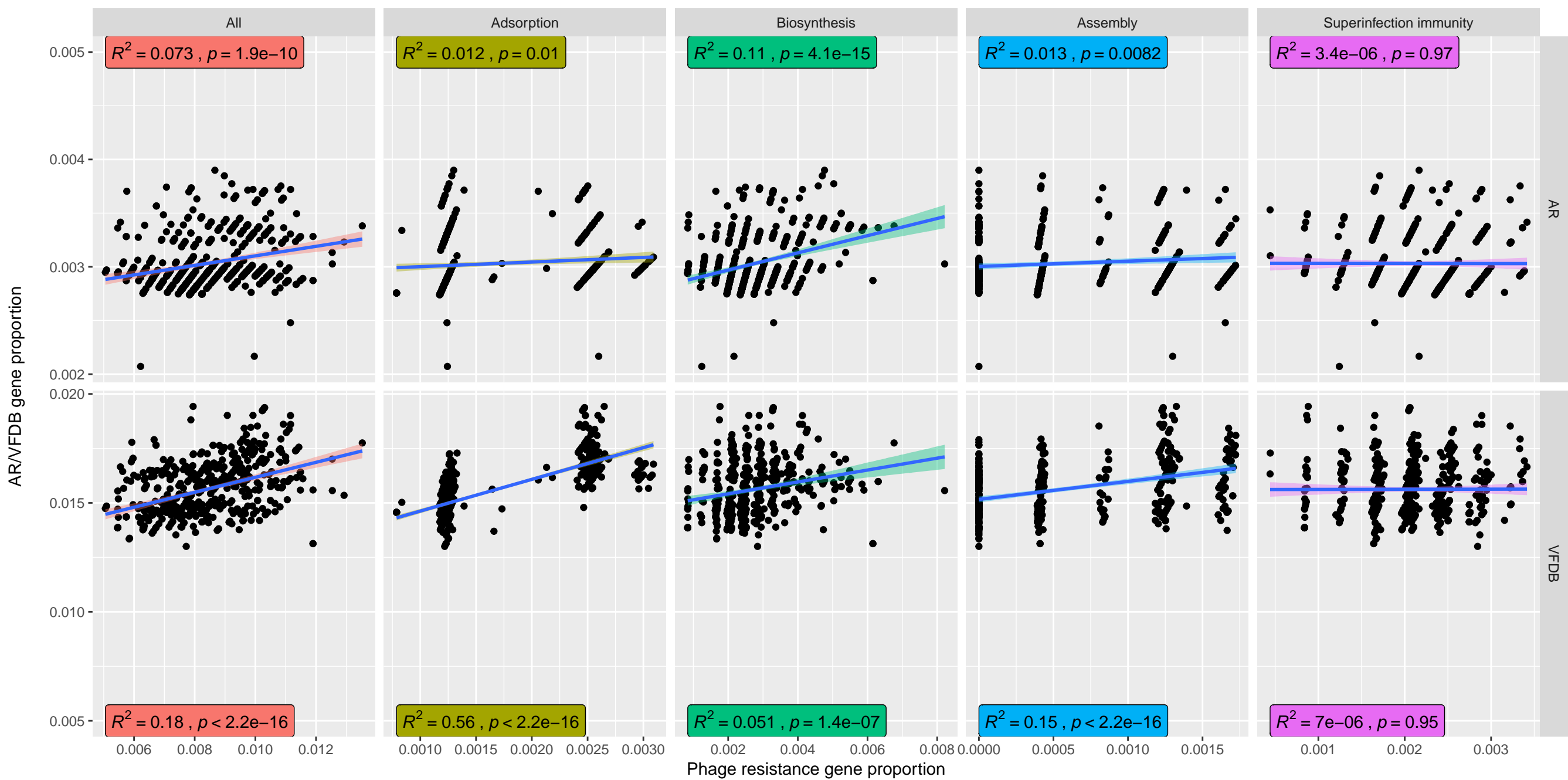

Supplement: FIG S7 [file msystems.01083-21-sf007.pdf]

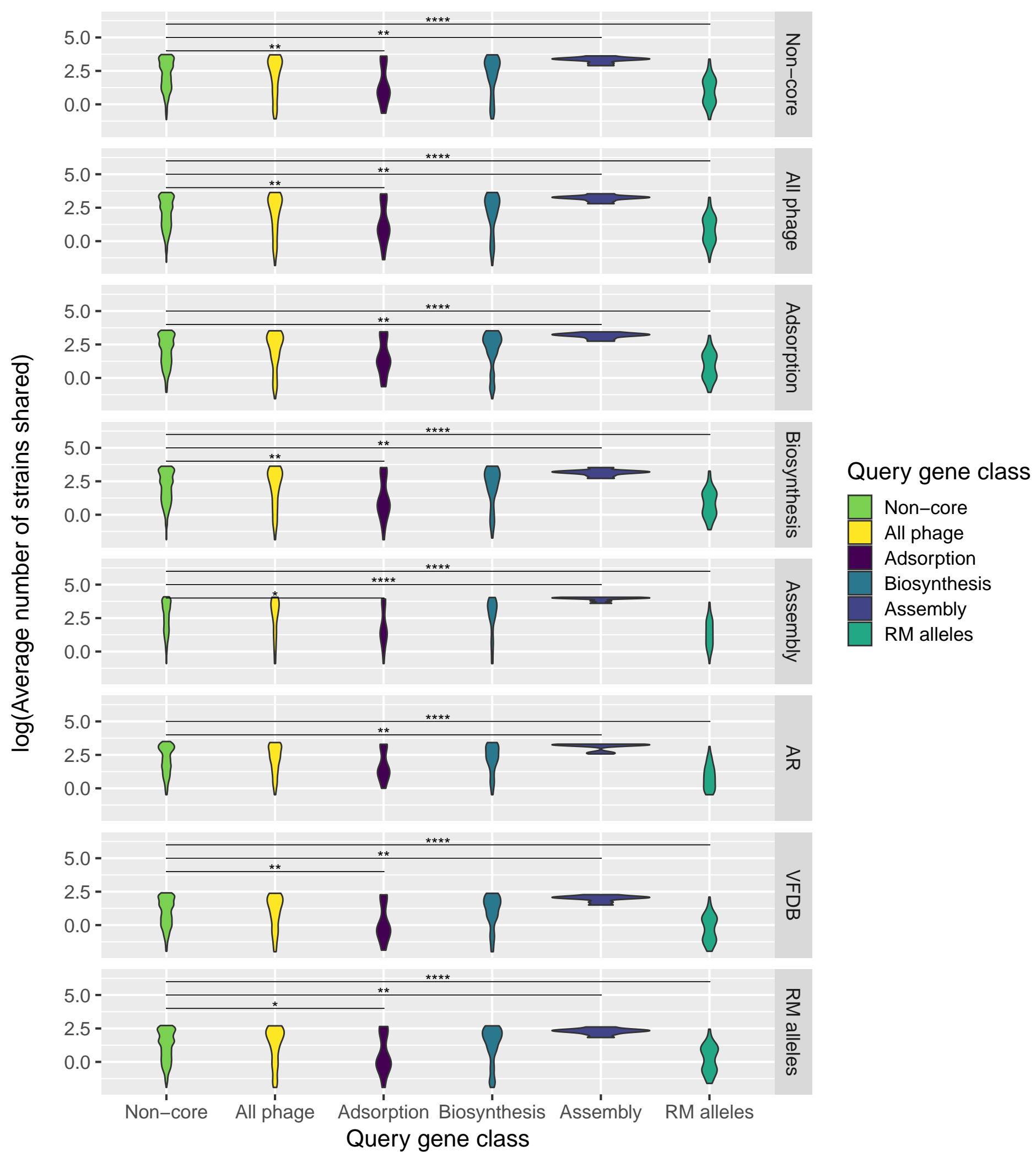

Supplement: FIG S8 [file msystems.01083-21-sf008.pdf]
