# Supplementary material for: Species-Scale Genomic Analysis of Staphylococcus aureus Genes Influencing Phage Host Range and Their Relationships to Virulence and Antibiotic Resistance Genes
Source: mSystems. 2022 Jan 18;7(1):e01083-21. doi: 10.1128/msystems.01083-21 (PMC8765062; doi:10.1128/msystems.01083-21)
Supplement: FIG S4 [file msystems.01083-21-sf004.pdf]

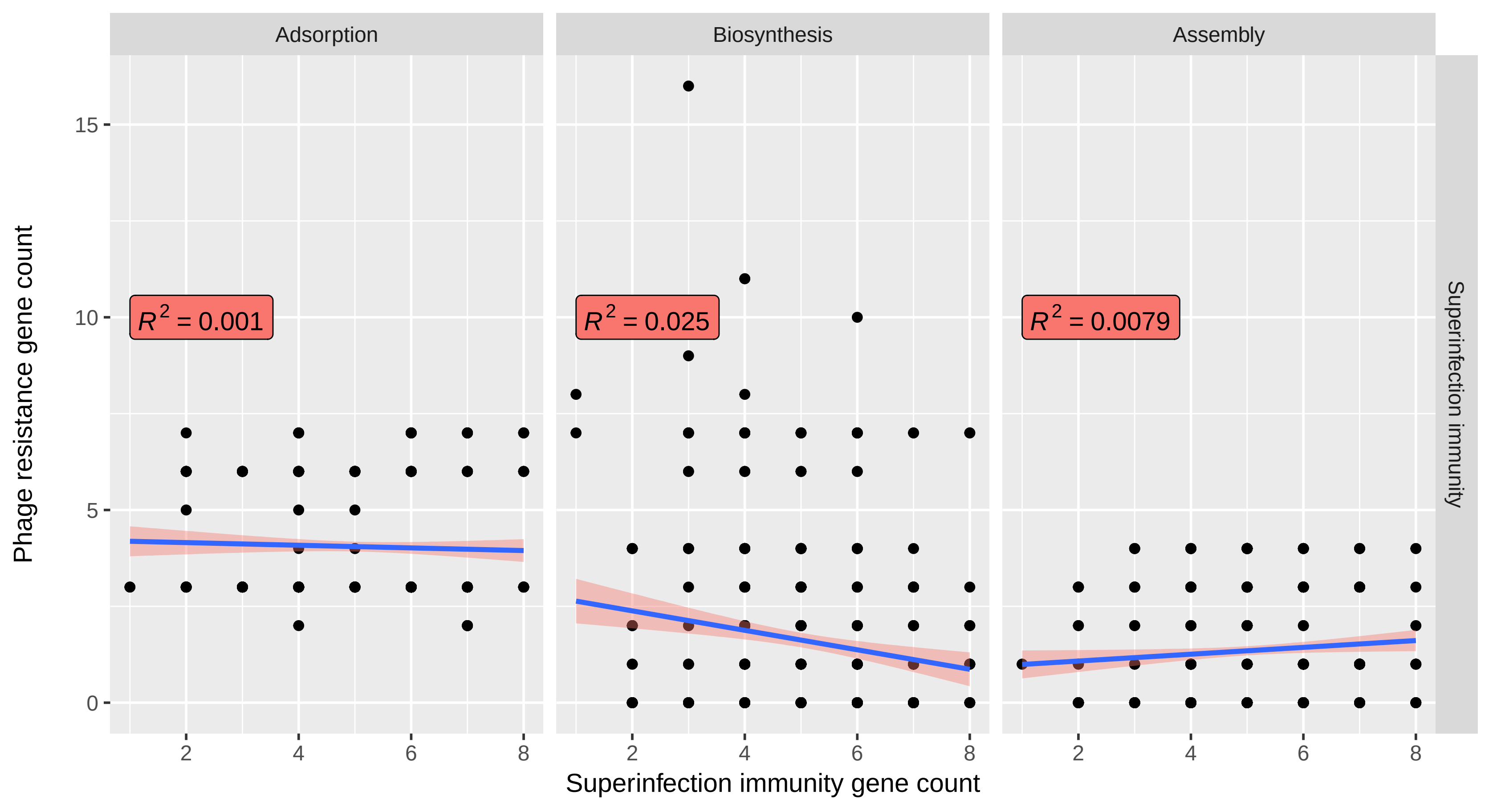

Adsorption
Biosynthesis
Assembly
Superinfection immunity
Phage resistance gene count
Superinfection immunity gene count
$R^2 = 0.001$
$R^2 = 0.025$
$R^2 = 0.0079$
0
5
10
15
2
4
6
8
